# A G protein alpha null mutation confers prolificacy potential in maize

**DOI:** 10.1093/jxb/erv215

**Published:** 2015-05-06

**Authors:** Daisuke Urano, David Jackson, Alan M. Jones

**Affiliations:** ^1^Department of Biology, The University of North Carolina, Chapel Hill, Coker Hall, NC 27599-3280, USA; ^2^Cold Spring Harbor Laboratory, Cold Spring Harbor, New York 11724, USA; ^3^Department of Pharmacology, The University of North Carolina, Chapel Hill, NC 27599-3280, USA

**Keywords:** Cell division, development, ear, G protein, maize, signalling.

## Abstract

Plasticity in plant development is controlled by environmental signals through largely unknown signalling networks. Signalling coupled by the heterotrimeric G protein complex underlies various developmental pathways in plants. The morphology of two plastic developmental pathways, root system architecture and female inflorescence formation, was quantitatively assessed in a mutant *compact plant 2* (*ct2*) lacking the alpha subunit of the heterotrimeric G protein complex in maize. The *ct2* mutant partially compensated for a reduced shoot height by increased total leaf number, and had far more ears, even in the presence of pollination signals. The maize heterotrimeric G protein complex is important in some plastic developmental traits in maize. In particular, the maize Gα subunit is required to dampen the overproduction of female inflorescences.

## Highlight

Maize COMPACT PLANT 2, the alpha subunit of the heterotrimeric G protein complex, is important for some plastic developmental traits: shoot and root system architectures, and ear production.

## Introduction

Maize, which originated in the Tehuacan Valley of Mexico, is a large grain plant and the most widely grown crop (Long and Fritz, 2001; Doebley, 2004). During domestication from its ancestor (teosinte), maize decreased the number of female inflorescences, while increasing inflorescence size, grain size and number (Doebley, 2004). Most modern maize lines, such as the B73 inbred line, initiate several ear branch shoots (shanks) per plant at successive main stalk nodes. Each shank has a single ear primordium at its tip and growth of this uppermost (‘top’ or ‘first’) ear suppresses the development of ears from lower nodes (second, third ears, etc.) on the main stalk (Pautler *et al.*, 2013; Wills *et al.*, 2013). Growth of the apical ear shoot also suppresses the formation of additional axillary ears on the same shank. In contrast to maize, development of productive ear shoots on branches from multiple nodes is common in teosinte, although it is occasionally observed in maize, where it is referred to as prolificacy (McClelland and Janssen, 1929; Frank and Hallauer, 1997; Moulia *et al.*, 1999). For example, under certain conditions, maize makes multiple ear shoots on the same shank or more rarely ‘twined’ ears, defined as two separate ears with separate husks at the same node (Frank and Hallauer, 1997). Successive nodes can also make productive (seed-bearing) ears, for example, this is common when plants are grown at lower densities. The frequency of prolificacy varies with genetic background and environmental factors, however, a molecular mechanism wiring the genetic and environmental factors remains poorly understood. In this paper, the involvement of the G protein signalling network in maize prolificacy has been reported, as well as its roles in shoot and root development.

Maize root and shoot architecture are examples of other plastic developmental traits (Brown *et al.*, 2011; Yu *et al.*, 2014). The juvenile maize seedling has a primary root and multiple seminal roots that originate from the subterranean embryo, whereas the adult plant also has crown roots emerging from aerial nodes. The overall architecture, specifically the number of each root type, lengths, and their position, is controlled by environmental cues such as the location and amount of water and nutrients in the soil profile. While maize root architecture is genetically encoded and some of these genes have been identified and studied (Hochholdinger and Tuberosa, 2009), little is known about how environmental signals are transduced to manifest root architecture (Casal *et al.*, 2004). Like roots, shoot architecture is similarly plastic, evident by observed changes that maximize energy capture in balance with water loss. For example, planting density has a major effect on shoot architecture (Ku *et al.*, 2015).

The heterotrimeric G protein complex, composed of α, β, and γ subunits, is an evolutionary conserved signalling complex that transmits signals from transmembrane receptors to intracellular proteins (Urano *et al.*, 2013). A null mutation of the maize Gα gene reduces shoot growth, leading to a dwarf phenotype, ear fasciation, and thicker tassel branches (Bommert *et al.*, 2013). Here, the nature of this phenotype has been explored in greater depth, with emphasis on the role of G protein signalling in ear development and prolificacy.

## Materials and methods

### Growth conditions

Seeds of wild-type B73 and the Gα-null mutant *ct2* (*ct2-ref*) (Bommert *et al.*, 2013) introgressed five generations into B73 were germinated and grown in 3″ soil pots for about 2 weeks in a greenhouse. The seedlings were transferred to 3-gallon pots having a diameter and height of 25cm each. The pots were placed on a water tray of 6cm in height filled with water two or three times a week. Nutrient was supplemented once a week beginning at the third week. Nutrient contents dissolved in tap water were 250 parts per million (ppm) of nitrogen, phosphate, and potassium, 0.63 ppm of magnesium and iron, 0.31 ppm of zinc and manganese, 0.16 ppm of copper and boron, and 0.06 ppm of molybdenum. Temperature was controlled within 25.3–28.1 °C (77.5–82.5 °F) in the day and 20.5–23.3 °C (69–74 °F) at night. Experiments were conducted between April and December 2014. On days when clouds reduced the ambient irradiation below 450W m^–2^, light was supplemented with 1000W high-intensity discharge lamps and these lamps were turned off when ambient light was above 900W m^–2^. Leaf stage (number of leaf collars), number of visible ears, height of leaf collars, and length and width of leaf blades were measured once a week. Plant height from the soil surface to the tip of the tassel was measured when tassels were fully developed.

### Root growth

B73 and *ct2-ref* seeds were germinated on soil for 6 d, then the seedlings were transferred to ¼× Murashige and Skoog (MS) media with 0.05% 2-(*N*-morpholino)ethanesulphonic acid as described previously (Urano *et al.*, 2014). The pH was adjusted to 5.7 with potassium hydroxide. The seedlings were grown in a 24h cycle chamber of 16h light at 210–220 μmol m^–1^ s^–1^ and 8h darkness at 28°C. The ¼× MS media was replaced with ½× MS media on the second week of hydroponics. The length of the longest crown root was measured on the 14th day after sowing seeds. The numbers of seminal and crown roots were counted on the 20th day.

### Statistical analyses

Data were analysed by the two-tailed Student’s *t* test between wild-type B73 and Gα-null *ct2* groups. Significant differences are shown with symbols of n.s. (not significant, *P* ≥0.05), * (*P* <0.05) or ** (*P* <0.01).

## Results and discussion

**Fig. 1.**
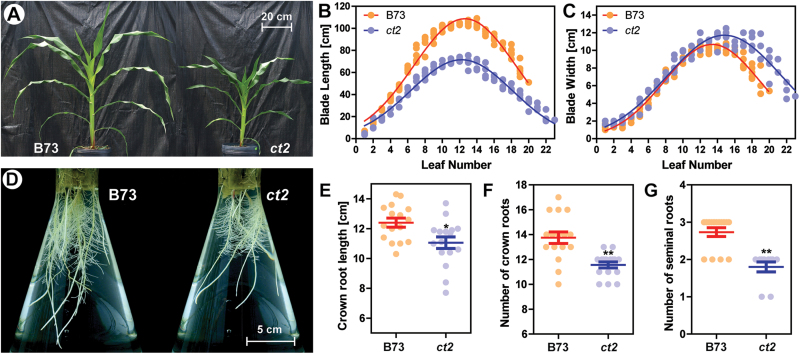
A Gα-null line decreases longitudinal growth in shoots and roots. (A) Five-week-old seedlings of B73 and the Gα-null *ct2* mutant. Scale bar=20cm. (B, C) Leaf length and width of B73 and *ct2*. Panels show raw values of B73 (orange dots, *n*=5) and *ct2* (blue dots, *n*=4) with a curve fitted by the Gaussian distribution function. (D) Representative roots of 16-d-old B73 and *ct2* seedlings grown in 2.0 l Erlenmeyer flasks. A scale shows 5cm. (E, F, G) The longest crown root length (E) was measured on the 14th day, and number of crown roots (F) and seminal roots (G) were measured on the 20th day. Panels show raw values of B73 (orange dots, *n*=16) and *ct2* (blue dots, *n*=16). Bars represent the means with standard errors of the mean. * or **, respectively, signifies a significant difference between B73 and *ct2* groups at the *P* value less than 0.05 or 0.01, by the two-tailed Student’s *t* test. n.s. signifies no significant difference at the *P* value of 0.05. Quantitated values are presented in Supplementary Table S1 at *JXB* online.

The leaf shape of the *ct2* mutants was noticeably different (Fig. 1A; 5-week-old plants of B73 and *ct2* grown in the greenhouse). The *ct2* mutation led to a shortening of the leaf blade by 18–42% in all leaves (Fig. 1B) and plant height by 32% (see Supplementary Fig. S1A, B at *JXB* online), while slightly increasing leaf width (Fig. 1C). The *ct2* plants also had an increased number of leaves per plant (B73, 18.4 leaves; *ct2*, 20.0 leaves), and a slightly delayed growth rate of leaves (see Supplementary Fig. S1C, D at *JXB* online). The total leaf area of B73 was 8369cm^2^ (*n*=5), compared with *ct2* total leaf area of 6515cm^2^ (*n*=4), 22% less than the wild type. Thus the increased number of leaves in *ct2* mutants did not fully compensate the reduced individual leaf area.

The *ct2* mutation also reduced root growth and crown root formation (Fig. 1D–G). Figure 1D shows B73 and *ct2* roots grown hydroponically for 2 weeks. The *ct2* mutant had fewer seminal roots, and fewer and shorter crown roots, as quantified in Fig. 1E–G. These results suggested that a Gα signalling network modulates cell proliferation both in shoots and roots, although the effect by Gα-null mutation was greater on the shoot than the root system (shoot, 32% reduction; root 11% reduction).

**Fig. 2.**
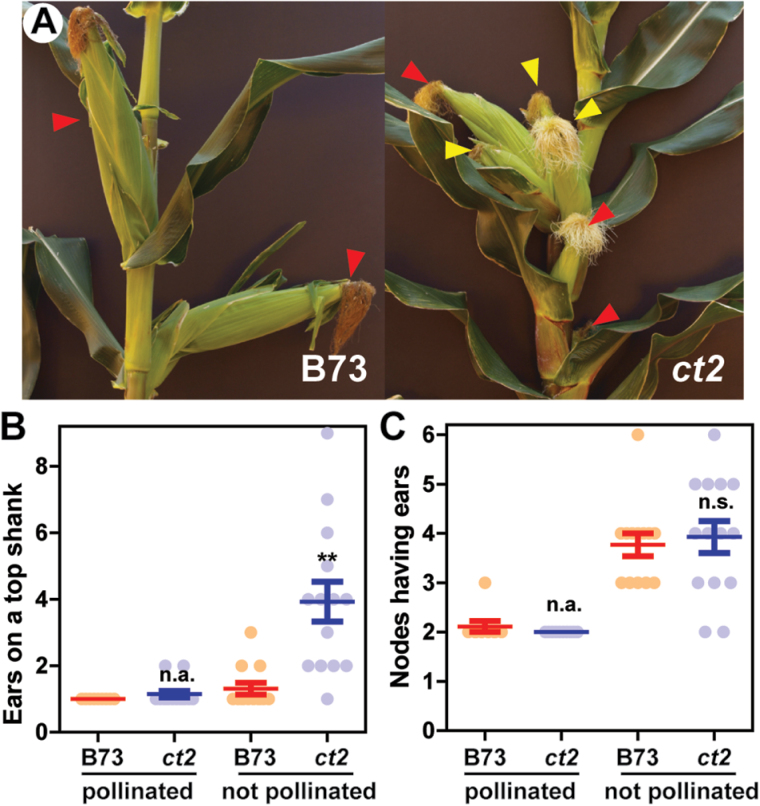
The *ct2* Gα-null mutant forms multiple ears at a single node. (A) The main stalk of unpollinated wild-type B73 and Gα-null *ct2* mutant. Red arrowheads point to apical ears with silks. Yellow arrowheads indicate axillary ears formed on the uppermost (top) ear shank. (B, C) Number of ears formed on the uppermost ear shank or on all nodes having ears. Data were collected from 15-week-old B73 and *ct2* plants. Graphs in (B) and (C) present raw values of B73 (blue dots) and *ct2* (orange dots), the means, and the standard errors. ** Represents significant difference between B73 and *ct2* groups at the *P* value less than 0.01 by the Student’s *t* test. n.s. signifies no significant difference at the *P* value of 0.05. n.a. Represents not statistically analysed, because all the values of the B73 or *ct2* group were identical. Quantitated values are available at Supplementary Table S2 at *JXB* online. See Supplementary Fig. S2 at *JXB* online for other images for wild-type B73 and Gα-null *ct2* plants.

In addition to the dwarf defect, we observed *ct2* plants having multiple ear shoots on a single shank (Fig. 2; see Supplementary Fig. S2 at *JXB* online). The axillary ear shoots were smaller and had poor kernel fill. Supplementary Fig. S2A, B at *JXB* online show representative stalks of B73 and *ct2* at the 14th week. Both B73 and *ct2* plants usually exhibited one or two visible ear shanks, each with a single ear at the apex, when the uppermost ear was pollinated. However, about 15% of *ct2* plants, while none of the B73 plants, formed several axillary ear shoots on the uppermost shank (Fig. 2B). Because poor kernel fill is associated with the multiple ear formation trait (McClelland and Janssen, 1929), pollination was inhibited and axillary ear formation was analysed (Fig. 2A; see Supplementary Fig. S2 at *JXB* online). While most B73 plants still exhibited a single ear on a shank under the non-pollinated condition, the uppermost ear node of *ct2* formed multiple visible axillary ears, as indicated by arrowheads (Fig. 2A). Figure 2B and Supplementary Fig. S2C, D at *JXB* online provide the quantitation of this phenotype. Therefore, *ct2* mutants, when unpollinated, had more visibly-developed ears per plant (B73, mean 4.1 ears; *ct2*, 7.8 ears), and on the uppermost node of the main stalk (B73, mean 1.3 ears; *ct2*, 3.9 ears). Inhibition of pollination similarly promoted development of ear shanks at lower nodes on the main stalk (Fig. 2C), however, no difference was observed between the B73 and *ct2* groups (B73, mean 3.8 nodes forming a visible ear shank; *ct2*, 3.9 nodes). Prolificacy was not observed in pollinated groups of B73 or *ct2* (Fig. 2B, C), suggesting that it requires both low pollination and mutation of *ct2*.

**Fig. 3.**
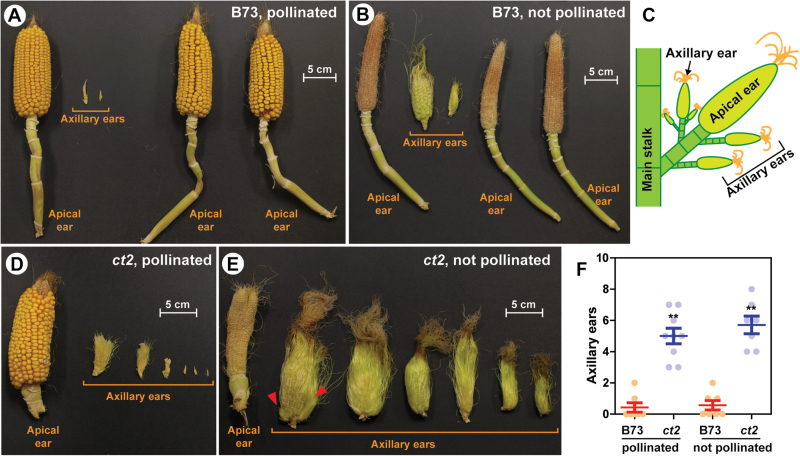
Female inflorescences formed on the uppermost shank. (A–E) Axillary ear shoots formed on the uppermost ear shanks of 15-week-old B73 or *ct2* plants with or without pollination. Apical and axillary ears are defined as shown in (C). Husk leaves were removed for imaging. (A, B) Apical and axillary ears sampled from three B73 plants. Note that axillary ears rarely emerged with the B73 genetic background. (D, E) Apical and axillary ears of a representative *ct2* plant. Red arrowheads point to secondary axillary branches emerging on an axillary ear shoot. Another image for *ct2* is presented in Supplementary Fig. S3 at *JXB* online. (F) Number of axillary ear shoots emerging on the uppermost shank of B73 and *ct2*. The graph shows raw values of B73 (blue dots) and *ct2* (orange dots), the means, and the standard errors. ** Signifies significant difference between B73 and *ct2* groups at the *P* value less than 0.01 by Student’s *t* test. Quantitated values are available at Supplementary Table S2 at *JXB* online.

Low pollination of *ct2* caused axillary ear formation two or more weeks after the apical ear emerged (see Supplementary Fig. S2C, E at *JXB* online), probably by releasing them from growth arrest. Because the *ct2* mutation showed an additive effect with low pollination, it was predicted that more female inflorescences were formed on *ct2* mutant shanks. Therefore, ear shoots were dissected and all mature and immature female inflorescences of B73 and *ct2* were counted (Fig. 3), and it was found that B73 had few axillary inflorescences (B73 with pollination, mean 0.43 axillary ears; B73 without pollination, 0.57 axillary ears) (Fig. 3F; see Supplementary Table S2 at *JXB* online). These axillary ear shoots aborted when the apical ear shoot was pollinated (Fig. 3A), but elongated when the apical ear had not been pollinated (Fig. 3B). The *ct2* mutant increased the number of axillary ear shoots (Fig. 3D–F) and occasionally exhibited secondary axillary ear shoots from the axillary ears (indicated by red arrowheads in Fig. 3E and in Supplementary Fig. S3 at *JXB* online). Pollination did not affect the number of inflorescences on the uppermost ear shank (*ct2* with pollination, mean 5.0 ears; *ct2* without pollination, 5.7 ears), but low pollination allowed them to elongate, as observed for B73 (Fig. 3B, E). These results indicate that the *ct2* mutation allowed more prolific formation of axillary ear shoots, while low pollination caused a general release of the axillary ear shoots from growth arrest.

**Fig. 4.**
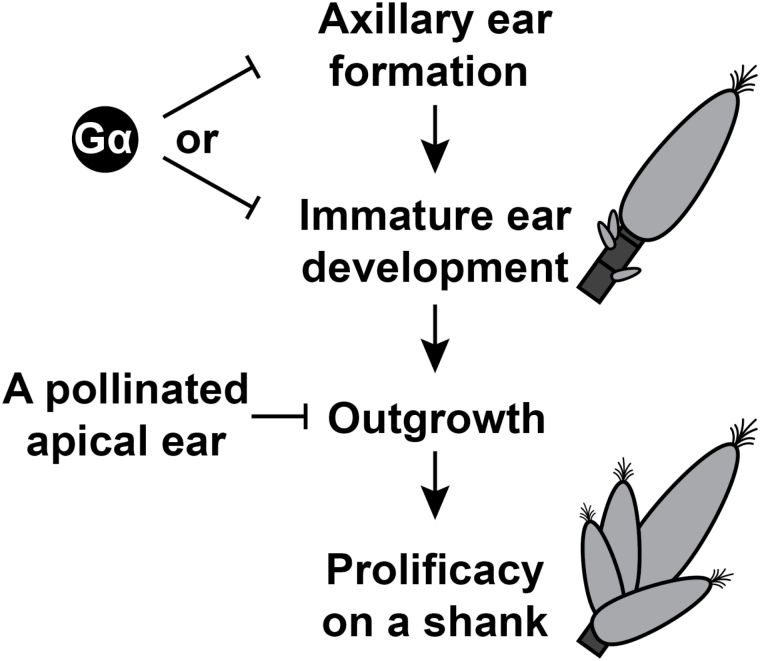
Proposed function of Gα and pollination signals on prolificacy. There are two sequential events for conferring prolificacy; a Gα-mediated axillary ear formation/development and a Gα-independent ear outgrowth. Domesticated maize intrinsically suppresses axillary ear formation on a shank. Activation of the Gα pathway represses axillary ear formation or immature ear development, while a pollinated-apical ear inhibits subsequent ear outgrowth perhaps through auxin or an unknown mediator. The latter pathway is independent of the Gα subunit.

Figure 4 shows a two-step model for conferring prolificacy. Genetics studies identified additional genes affecting the ear formation trait. Activation of a transcription factor, *BARREN STALK1* (*BA1*), initiates the axillary ear shoot meristems, while another transcription factor gene, *GRASSY TILLERS1* (*GT1*), suppresses the outgrowth of immature inflorescences (Ritter *et al.*, 2002; Gallavotti *et al.*, 2004; Whipple *et al.*, 2011). A different expression profile of *GT1* in the nodal plexus probably caused a distinct ear branching pattern between maize and teosinte (Wills *et al.*, 2013). Our results prompt the speculation that the G protein network regulates axillary meristem initiation/transition of axillary buds to reproductive development and/or outgrowth of immature ear shoots (Fig. 4), so may control these transcription factors. Although the signalling mechanism regulating these and other genetic components remains poorly understood, it is empirically known that ear outgrowth requires ample energy resources—water, light, and nutrients (Lejeune and Bernier, 1996; Moulia *et al.*, 1999; Markham and Stoltenberg, 2010). Multiple hormones—auxin, cytokinin, and strigolactones—also regulate the dormancy of axillary buds (Pautler *et al.*, 2013). Maize and other plant G-protein networks couple those extracellular stimuli and modulate meristem activity, cell proliferation, and cellular senescence (Bommert *et al.*, 2013; Urano *et al.*, 2013; Sun *et al.*, 2014), therefore G protein signalling may bridge these extracellular signals to ear development and outgrowth. The phenotypes described here, including root system and ear shoot architecture,are obvious plastic traits in plant development that have been selected during crop domestication and our results suggest that G protein signalling networks modulate the expression of these key agronomic traits. It will also be interesting to ask how natural variation in G protein signalling components has contributed to crop improvement.

## Supplementary data

Supplementary data can be found at *JXB* online.

Supplementary Fig. S1. Vegetative growth of B73 and Gα-null *ct2* lines.

Supplementary Fig. S2. Ear formation of B73 and Gα-null *ct2* lines.

Supplementary Fig. S3. Apical and axillary ears of a representative *ct2* plant.

Supplementary Table S1. Shoot and root growth of B73 and Gα-null *ct2* lines.

Supplementary Table S2. Female inflorescence formation in B73 and Gα-null *ct2* lines.

Supplementary Data
