# Supplementary material for: A G protein alpha null mutation confers prolificacy potential in maize
Source: J Exp Bot. 2015 May 6;66(15):4511–5. doi: 10.1093/jxb/erv215 (PMC4507758; doi:10.1093/jxb/erv215)
Supplement: Supplementary Data [file supp_66_15_4511__index.html]

A G protein alpha null mutation confers prolificacy potential in maize — A G protein alpha null mutation confers prolificacy potential in maize — Supplementary Data 

# A G protein alpha null mutation confers prolificacy potential in maize

## Supplementary Data

Data files

**Files in this Data Supplement:**

- Supplementary Data - Supplementary Data
