# Supplementary material for: A G protein alpha null mutation confers prolificacy potential in maize
Source: J Exp Bot. 2015 May 6;66(15):4511–5. doi: 10.1093/jxb/erv215 (PMC4507758; doi:10.1093/jxb/erv215)
Supplement: Supplementary Data [file supp_erv215_jexbot146191_file001.pdf]

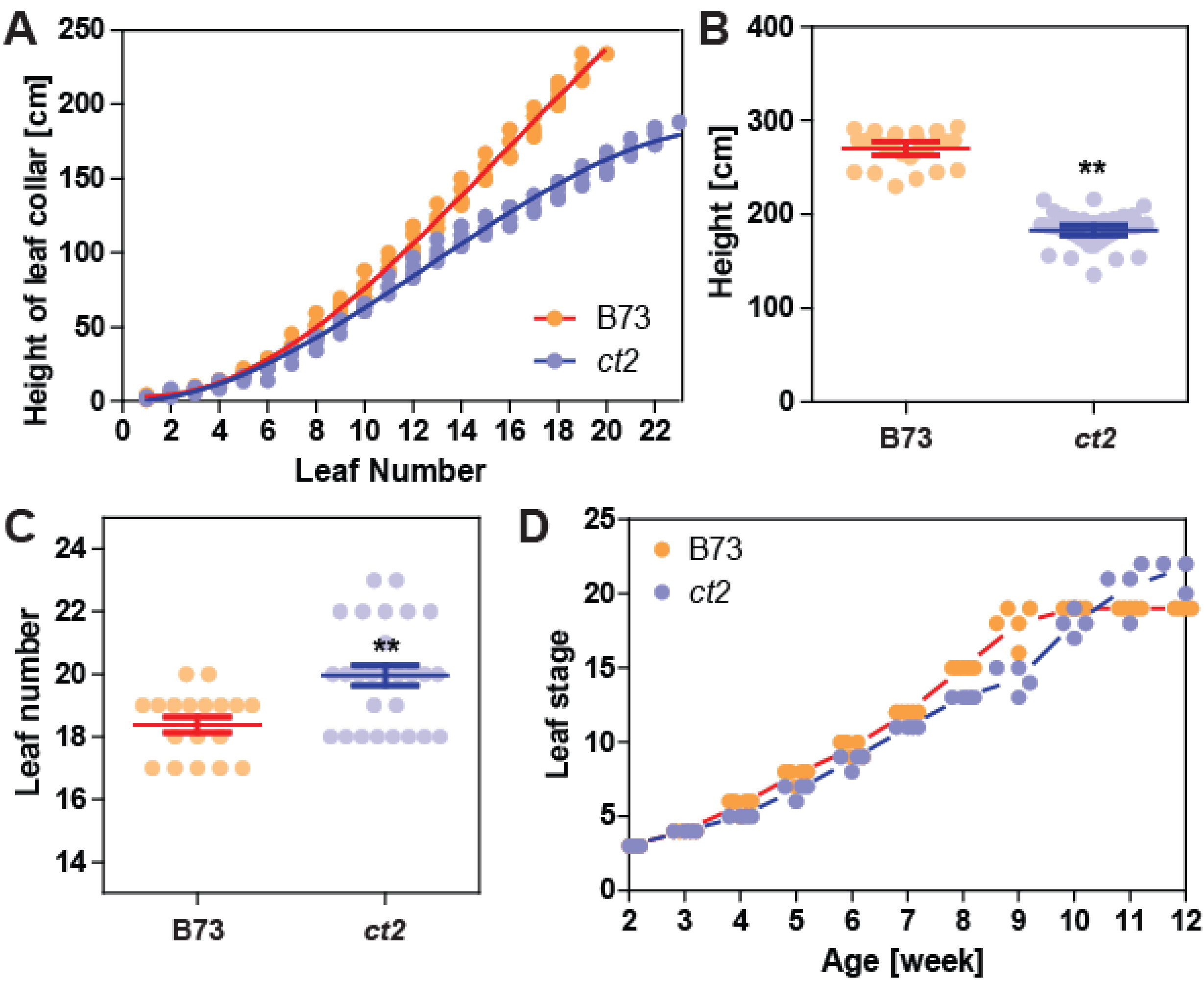

Figure S1. Vegetative growth of B73 and Gα-null *ct2* lines

(**A**) Height of leaf collars from the soil surface. The graph shows the raw values of B73 (orange dots, n = 5) and *ct2* (blue dots, n = 4) with a curve fitted by a third-order polynomial function. (**B**) Plant height from the soil surface to a tassel tip of B73 (n = 26) and *ct2* (n = 42). (**C**) Total leaf number of B73 (n = 18) and *ct2* (n = 27). The B and C panels plot the raw data with the thin bars representing means and the error bars representing the standard error of the mean. ** signifies a statistically significant difference between B73 and *ct2* at the p value less than 0.01 according to the two-tailed Student's t-test. (**D**) Number of leaves observed over an eleven week period. The graph shows values of B73 (orange dots, n = 5) or *ct2* (blue dots, n = 4) with a line connecting the mean values. Quantitated values for the panels B and C are presented in Table S1.

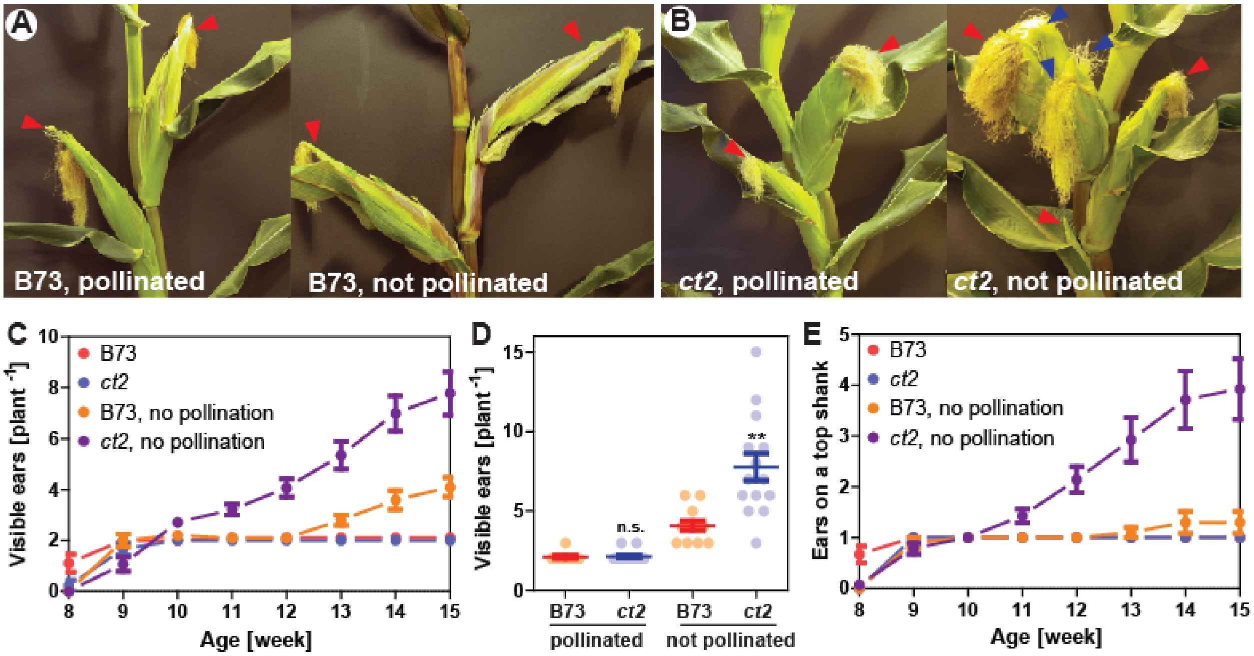

Figure S2. Ear formation on B73 and Gα-null *ct2* lines

(**A, B**) Ear shank images of B73 (**A**) or *ct2* (**B**) with or without pollination. Images show representative main stalks of 14-week old plants. Red arrowheads indicate apical ears with visible silks. Blue arrowheads indicate axillary ears formed on the top shank. (**C**) Number of visible ears per plant. The panel shows the mean values of B73 with pollination (red dots, n = 9), *ct2* with pollination (blue dots, n = 9), B73 without pollination (orange dots, n = 10) and *ct2* without pollination (purple dots, n = 14) over an eight week period. Error bars represent SEM. (**D**) Number of visible ears on 15-week old B73 and *ct2* plants. The graph plots raw values with bars representing the means and error bars representing the standard error of the mean. Quantitated values are presented in Table S2. (**E**) Number of ears formed at the uppermost node over an eight week period. Symbols, sample numbers, and error bars are the same as for C.

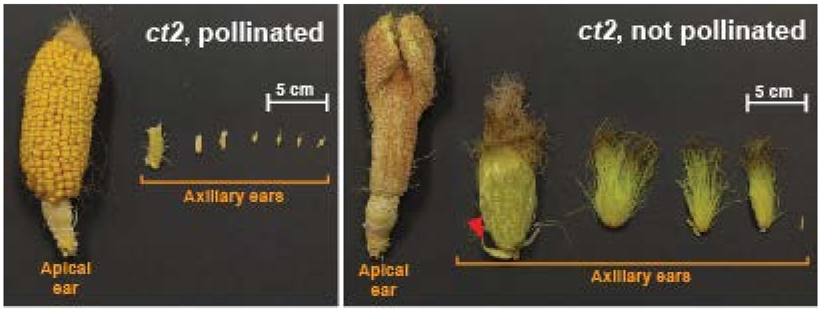

Figure S3. Apical and axillary ears of a representative *ct2* plant.

Uppermost ear shanks of 15-week old *ct2* plants with or without pollination. Husk leaves were removed for imaging. A red arrowhead indicates a secondary axillary branch emerging on an axillary ear. Another image is presented in Fig. 3.

Table S1. Shoot and root growth of B73 and Gα-null *ct2* lines

Height of plants (length from soil surface to a tassel tip) and the number of leaves were measured for wild type (B73) or Gα-null (*ct2*) plants grown in a greenhouse. Length of the longest crown root was measured for B73 and *ct2* grown hydroponically for 14 days. Number of seminal or crown roots were measured on the 20th day. Data represent means with the standard error. Numbers in parentheses represent the number of seedlings quantitated. * or ** respectively signifies a significant difference between B73 and *ct2* groups at the p value less than 0.05 or 0.01 (the two-tailed Student's t-test). Raw values are as plotted in figure 1E, 1F, 1G, S1B and S1C.

	Height of plants [cm]	Number of leaf collars	Length of crown roots [cm]	Number of crown roots	Number of seminal roots
B73	270.4 ± 3.5 (26)	18.4 ± 0.2 (18)	12.4 ± 0.3 (16)	13.8 ± 0.5 (16)	2.7 ± 0.1 (16)
ct2	183.3 ± 2.6 (42) **	20.0 ± 0.3 (27) **	11.0 ± 0.4 (16) *	11.6 ± 0.2 (16) **	1.8 ± 0.1 (16) **

Table S2. Female inflorescence formation in B73 and Gα-null *ct2* lines

B73 or *ct2* plants were grown in a greenhouse with or without pollination. The 15-week old plants were quantitated for numbers of visible ears per plant, visible ears on an uppermost shank, ear shanks formed from a main stalk and axillary ear shoots developed on the uppermost shank. Data represent means, the standard errors, and the number of measured samples. ** signifies a significant difference between B73 and *ct2* groups at the p value less than 0.01 (the two-tailed Student's t-test). n.s. signifies no significant difference at the p value of 0.05. n.a. means not statistically analyzed, because all values of the B73 or *ct2* group were identical. Raw values are as plotted in figure 2B, 2C, 3F and S2D.

		Ears per plant	Ears on the uppermost shank	Ear shanks formed from the main stalk	axillary female inflorescences
B73	pollinated	2.11 ± 0.11 (9)	1.00 ± 0.00 (9)	2.11 ± 0.11 (9)	0.43 ± 0.30 (7)
	not pollinated	4.08 ± 0.29 (13)	1.31 ± 0.17 (13)	3.77 ± 0.23 (13)	0.57 ± 0.30 (7)
ct2	pollinated	2.14 ± 0.10 (14) n.s.	1.14 ± 0.10 (14) n.a.	2.00 ± 0.00 (14) n.a.	5.00 ± 0.50 (9) **
	not pollinated	7.79 ± 0.85 (14) **	3.93 ± 0.60 (14) **	3.93 ± 0.32 (14) n.s.	5.71 ± 0.57 (7) **
